# The Parental Blueprint: Early Childhood Lifestyle Habits and Family Factors Predict Opting for Middle Childhood Sport Involvement

**DOI:** 10.3390/children12060732

**Published:** 2025-06-03

**Authors:** Stéfanie Routhier-Guilmette, Kianoush Harandian, Sophie-Anne Turcotte, Nairy Kazandjian, Matteo Privitera, Linda S. Pagani

**Affiliations:** 1 Department of Psychology, University of Montreal, Montreal, QC H3C 3J7, Canada; stefanie.routhier-guilmette@umontreal.ca; 2 School of Psycho-Education, University of Montreal, Montreal, QC H3C 3J7, Canada; kianoush.harandian@umontreal.ca (K.H.); sophie-anne.turcotte@umontreal.ca (S.-A.T.); nairy.kazandjian@umontreal.ca (N.K.); 3 School Environment Research Group (GRES), Montreal, QC H3N 1X7, Canada; 4 Sainte-Justine’s Pediatric Hospital Research Center, University of Montreal, Montreal, QC H3T 1C5, Canada; 5 Department of Public Health, Experimental Medicine and Forensic Sciences, University of Pavia, 27100 Pavia, Italy; matteo.privitera01@universitadipavia.it; 6 Laboratory of Human Locomotion, University of Pavia, 27100 Pavia, Italy; 7 Department of Brain and Behavioral Sciences, University of Pavia, 27100 Pavia, Italy

**Keywords:** early childhood, lifestyle habits, sport participation, screen time, child development, longitudinal study

## Abstract

Background/objectives: Early childhood is marked by rapid physical and cognitive development, underscoring the critical influence of parental choices in shaping habits, pastimes, and traditions. The World Health Organization highlights habits of physical activity, sleep, and screen use as modulators of healthy development and well-being in young children. As a first, this birth cohort study investigates the relationship between movement behaviors in toddlerhood and subsequent sport participation during middle childhood. Methods: Participants are 930 boys and 926 girls from the Quebec Longitudinal Study of Child Development. Using a population-based analytical approach, we examined the relationship between parent-reported active leisure, sleep, and sedentary screen time at age 2 years and trajectory of sport participation between ages 6 and 10 years using sex-stratified linear regressions that account for a range of pre-existing individual and family confounding factors. Results: Many child and family characteristics predicted early childhood movement behaviors, distinctly for boys and girls. Moreover, parental regulation of screen time during early childhood significantly influenced their decisions to enroll and encourage sport participation in sons during school age. Specifically, boys who spent more than 1 h/day on screens had 10% fewer chances of consistently participating in sport throughout middle childhood, even after accounting for adherence to other lifestyle habits. Conclusions: Our findings suggest that parental values regarding early childhood screen exposure are an effective potential target for intervention fostering healthy and active development. Future research should focus on parent and child motivations for sport involvement in the long-term.

## 1. Introduction

The first thousand days represent a key period of human development, in which rapid strides in physical, cognitive, and socio-emotional growth lay foundation for a lifetime of learning and well-being [1]. To support healthy development, the World Health Organization (WHO) [2] has provided early childhood guidelines on movement and rest which emphasize the importance of reducing sedentary screen time, encouraging active play, and ensuring adequate sleep to promote healthy development.

There are three features which comprise a healthy early childhood lifestyle [3]. First, toddlers should engage in at least 180 min of physical activity in various formats at any intensity throughout the day. As they become preschoolers in the third and fourth year of life, that 180-min period should have at least 60 min of moderate-to-vigorous intensity activity. Second, to diminish sedentariness and increase essential psychological stimulation and social interaction, screen time should be limited from infancy onward. Specifically, the WHO recommends no screen time until age 2 years and no more than 1 h daily for older toddlers (with less time preferred). Finally, a 24-h day should comprise 11 to 14 h of good quality sleep, including napping time, for children aged 1 to 2 years, and 10 to 13 h for children aged 3 to 4 years. 

Parental knowledge of the importance of physical activity, sleep, and limited screen time for optimal child developmental outcomes remains limited [4]. In a Canadian study, very few preschool children (13%) met all three recommendations, according to parent reports [4]. Only one-fifth met the screen time guidelines [5]. An investigation on New Zealand preschoolers, using accelerometer data, found similar results [6]. Likewise, a recent large-scale Australian study found that only one-fifth of toddlers met all three guidelines, with less than a third of children adhering to screen time guidelines [7]. In fact, children who met all three guidelines at a given age were more likely to meet all three guidelines at a subsequent age [8]. Therefore, literature indicates that despite the well-documented benefits of physical activity and healthy habits, features of an optimal early childhood lifestyle can vary widely among families.

Taken together, these studies suggest that key habits in early childhood set the stage for subsequent lifestyle characteristics. Parents have a lot to do with choices about how children spend their time [4]. For instance, active leisure during family time, enforcement of sleep schedules, and being aware of daily screen time are typical parental responsibilities that begin in early childhood [6].

Research indicates that active preschool children who sleep enough and have limited screen time experience optimal developmental benefits across multiple domains, including improved physical health, cognitive function, and socio-emotional well-being [9]. While some lifestyle features of early childhood movement and rest naturally align with optimal developmental habits, the environmental and behavioral factors that drive this alignment remain unclear. Furthermore, relationships between early childhood active leisure, screen, and sleep habits and subsequent parental choices about middle childhood extracurricular activities warrant empirical investigation.

From a theoretical perspective, early childhood is deeply embedded within a complex ecological system and represents a sensitive period during which caregivers play a key role in shaping children’s lifestyle behaviors [10,11]. Bronfenbrenner’s ecological systems theory emphasizes the dynamic interplay between children and their surrounding environments, including family context, where parental modeling, household routines, and values significantly shape child behavior [12]. During such periods, exposure to structured health habits can have enduring effects on their long-term behaviors and play a crucial role in shaping lifestyle predispositions [1,13,14]. 

Social cognitive theory posits that children acquire and internalize behaviors by observing and imitating significant others, particularly parents [15]. Through mechanisms such as reinforcement and observational learning, children learn to associate health behaviors with social norms and familial expectations. Consequently, sedentary habits and screen time usage are generally influenced by child-rearing practices and family activities [4]. Additionally, parenting style topologies, such as authoritative, permissive, or neglectful approaches, can moderate consistency, structure, and emotional tone of health-related routines [16].

Within this framework, excessive screen time, limited structured physical activity, and poor sleep habits may reflect broader parenting behaviors or environmental constraints that hinder optimal development. For instance, permissive parenting fails to establish clear limits around sedentary behavior, while authoritarian approaches may enforce schedules but reduce child intrinsic motivation to engage in healthy activities [16]. Such theoretical lenses provide valuable insight into how systems influence early habits and, in turn, shape future patterns of active or sedentary living [17].

Extant research has largely overlooked how parental choices of childhood lifestyle impact long-term physical activity habits of children [18]. Most studies focus on short-term interventions or experimental designs, failing to capture the natural ways in which parents integrate or neglect these recommendations in everyday life. Moreover, the predominance of cross-sectional studies limits our ability to examine long-term relationships. Such snapshot designs obscure the true developmental trajectories of active or sedentary behaviors due to the influence of confounding variables [19]. To address this gap, longitudinal birth cohort data provide an opportunity to examine relationships between naturally occurring early childhood movement, screen time, and sleep habits and subsequent parental choices about middle childhood extracurricular activities. Another limitation of previous research is the tendency to treat child sex as a covariate, resulting in findings that discount biological influences and contextual experiences [20]. However, this approach does not accurately reflect the reality that boys and girls are exposed to unique risk and protective factors driven by both biological and environmental influences [21,22]. Therefore, parents socialize and educate their children according to gender norms, which can significantly impact choices toward a healthy lifestyle and long-term active leisure habits [23].

Using a population-based birth cohort of boys and girls born between 1997 and 1998, this study aims to investigate prospective associations between lifestyle factors in toddlerhood and sport participation in middle childhood. Specifically, we investigated whether active leisure, screen time, and sleep at age 2 years predict subsequent extracurricular sport trajectories between ages 6 to 10 years in typically developing boys and girls. We examine whether individual and combined early childhood lifestyle habits predict subsequent sport participation in middle childhood and acknowledge that such participation largely reflects parental decisions in toddlerhood. As a natural experiment, our objective informs how lifestyle behaviors in toddlers, individually or jointly, shape later sport participation. We hypothesized that optimal early lifestyle characteristics would predict consistent youth sport participation, highlighting the lasting impact of parental choice and early child-rearing on active leisure habits.

## 2. Materials and Methods

### 2.1. Participants and Design

A sample of children born between 1997 and 1998 in Quebec, Canada, was selected from the Quebec Longitudinal Study of Child Development (QLSCD) coordinated by the *Institut de la statistique du Québec*. A randomized, regionally stratified sampling method was used to select 2837 newborns. In total, 82% of the children (2120 participants) were included and followed with their families starting at 5 months of age, after obtaining parental consent and verifying inclusion criteria. Among those not retained, 93 were from First Nations communities, 186 were untraceable, and 438 did not provide parental consent to participate. This IRB-approved study collected data through interviews and self-reported questionnaires annually in early childhood and biennially during school-age years. A sample of 930 boys and 926 girls with complete data on movement behaviors at age 2 years was retained for the prospective-longitudinal design.

### 2.2. Measures: Toddler Lifestyle Predictor Variables (Age 2 Years)

*Screen Time.* Parents reported the amount of time their child spent: (a) watching television programs, (b) watching videos, (c) using a computer, and (d) playing video games during a structured interview. Total screen time, expressed in hours and minutes for an average day, was calculated by summing up these four activities. Sedentary screen time was then dichotomized into two categories: (0) more than one hour per day and (1) one hour or less per day.

*Sleep.* Mothers reported average child sleep duration (in hours and minutes), including daytime naps, using a self-administered questionnaire. Sleep duration was classified as (0) less than 11 h or more than 14 h, and (1) between 11 and 14 h.

*Active Leisure.* A structured interview was conducted in which parents reported the frequency, over the past 12 months of engaging in sport, hobbies, and games with their child. Response options, based on a Likert scale, ranged from (1) never to (5) several times a day. Scores were dichotomized into (0) less than once a day and (1) at least once a day.

*Combined Daily Movement Behaviors.* The reported behaviors were combined into an index designed to capture an integrative approach to daily lifestyle during early childhood, inspired by WHO guidelines on physical activity, sedentary behavior, and sleep [2]. This index assigned scores ranging from (0) no adequate behaviors to (3) presence of all three recommended behaviors.

### 2.3. Measures: Outcome Variable (Ages 6 to 10 Years)

*Sport Participation Trajectory.* Child participation in extracurricular sport over the past 12 months was reported by the mother at ages 6, 7, and 8 years. Two items were used: “In the last 12 months, outside of school hours, how often has your child: (a) Taken part in sport with a coach or instructor (except for dance or gymnastics courses?) and (b) taken organized physical activity lessons or classes such as dance, gymnastics, martial arts, or circus arts?” Both items were rated on a Likert scale (0 = never, 1 = roughly once a month, 2 = roughly once a week, 3 = several times a week, 4 = roughly every day, 5 = one session, 6 = two sessions). “One session” or “two sessions” may be rated higher than “every day” because they imply more structured, intensive participation, even if less frequent. Mothers also reported on child extracurricular sport participation across 3 items at age 10 years: “How many times a week has your child participated: (a) In organized sport or physical activity with a coach or instructor last summer?, (b) in organized sport or physical activity with a coach at school since last September, outside of physical education classes?, and (c) in organized sport or physical activity with a coach outside of school since last September?” (0 = never, 1 = less than once a week, 2 = once a week, 3 = twice a week, 4 = three times a week, 5 = four times a week, 6 = five times a week or more). All items were recoded as: 0 = no participation and 1 = any participation. Based on this data, Brière et al. [23] identified two distinct patterns of sport participation trajectories through Growth Mixture Modelling: (0) low-inconsistent sport participation and (1) consistent sport participation. Previous studies have demonstrated the predictive validity of these trajectories [24,25].

### 2.4. Measures: Pre-Existing and Concurrent Confound Controls (Ages 5 Months to 2 Years)

Individual and family factors prior to or in toddlerhood were statistically considered, enabling the adoption of a prospective design that better alludes to causal interpretations based on longitudinal data. The following factors were controlled as potential confounding variables.

*Individual child variables* included the child body mass index (BMI), measured by trained examiners at age 17 months (0 = below the median, 1 = above the median), child difficult and unpredictable temperament as perceived by parents, based on items from the Infant Characteristics Questionnaire (ICQ) at age 17 months (20 items, **α** = 0.83; 0 = below the median, 1 = above the median) [26,27], and parent perception of child physical activity at age 2 years (0 = more active or similarly active to others, 1 = less active than others).

*Family variables* include self-reported maternal depressive symptoms on an abridged version of the Center for Epidemiologic Studies Depression Scale (13 items, **α** = 0.81; 0 = below the median, 1 = above the median) [28,29], maternal education (0 = finished high school, 1 = did not finish high school), and parent antisocial antecedents using the National Institute of Mental Health-Diagnostic Interview Schedule assessment at age 5 months (17 items, **α** = 0.61; 0 = below the median, 1 = above the median) [30]. These also included parent-reported family dysfunction based on the McMaster Family Assessment Device (9 items, **α** = 0.84; 0 = below the median, 1 = above the median) [31,32] and self-reported maternal BMI at age 17 months (0 = below the median, 1 = above the median), as well as parent-reported family configuration (0 = intact, 1 = non-intact) and family income at age 2 years (0 = sufficient income, 1 = insufficient income), as defined by the Canadian low-income cut-off of that year provided by Statistics Canada [33]. The questionnaires used have all been validated [26,27,28,29,30,31,32].

### 2.5. Data Analytic Strategies

First, we generated descriptive statistics using SPSS (v.29). Then, we used least squares multiple regressions, stratified by sex, to estimate long-term prospective linear associations between lifestyle factors at age 2 years and subsequent youth sport participation trajectories from ages 6 to 10 years established from latent class growth analysis in Brière et al. [23]. To make the design truly prospective, we accounted for potential pre-existing and concurrent family and individual variables that could potentially confound or compete with our hypothesis in our long-term estimates. Statistical control of such potential associations helps isolate estimates between our predictor and outcome and addresses potential omitted variable bias.

Multiple imputations were conducted to account for possible response and attrition bias. Our analyses were conducted with five imputed iterations. All variables were included in the imputation model, with predictors and control variables specified as independent. To assess the robustness of the analyses, imputation summaries were reviewed. It is noteworthy that the analyses conducted on the original non-imputed subsample and the subsample corrected for response and attrition bias yielded similar results.

## 3. Results

Table 1 reports descriptive statistics of participants based on the predictors, outcome, and individual and family characteristics. At age 2 years, approximately one-quarter of boys and girls spend less than one hour per day in front of a screen and 78% of children, regardless of sex, slept an average between 11 and 14 h per day. Regarding participation in active leisure activities, 33% of boys and 35% of girls engaged in such activities daily. Overall, most children adequately followed one or two recommended lifestyle behaviors, while 11.9% of boys and 11.1% of girls did not adhere to any, and only 7.4% of boys and 8.1% of girls met all three recommendations daily. Moreover, 62.4% of boys and 65.8% of girls maintained a stable trajectory of sport participation through middle childhood, as opposed to a low-inconsistent trajectory.

Table 2 reports the adjusted unstandardized regression coefficients with standard errors, reflecting associations between individual and family preexisting and concurrent characteristics from ages 5 months to age 2 years and movement behaviors at age 2 years, stratified by sex. Sons of mothers exhibiting depressive symptoms were 8% less likely to engage in daily active leisure activities (*p* ≤ 0.05) and showed a 10% unit decrease in overall movement behavior involvement (*p* ≤ 0.05). Boys with a BMI above the median, as well as those with a more difficult temperament, were 10% (*p* ≤ 0.01) and 8% (*p* ≤ 0.05) less likely to obtain recommended amounts of sleep per day, respectively. Girls whose mothers did not complete high school had a 12% higher likelihood of sleeping less than 11 h per day or more than 14 h (*p* ≤ 0.01) and showed a 13% unit decrease (*p* ≤ 0.05) in adherence to adequate combined movement behaviors, whereas girls whose mothers exhibited greater depressive symptoms were 10% more likely to spend more than an hour per day on screens (*p* ≤ 0.01).

At age 17 months, girls with a higher body mass index were 13% more likely (*p* ≤ 0.001) to spend more than an hour per day on screens and showed 12% unit decreases (*p* ≤ 0.01) in overall movement behavior involvement. Girls living in dysfunctional families were 12% (*p* ≤ 0.01) more likely to spend less than one hour per day on screens, but 12% (*p* ≤ 0.01) less likely to participate in active leisure activities every day. Those girls from families with income below the poverty line were 11% more likely to spend over an hour per day on screens (*p* ≤ 0.05) and 10% more likely to sleep less than 11 h or more than 14 h per day (*p* ≤ 0.05). They also demonstrated 9% unit decreases (*p* ≤ 0.05) in overall movement behavior involvement.

Table 3 reports the adjusted unstandardized regression coefficients, representing the associations between screen time, sleep, and active leisure at age 2 years and sport participation trajectories between ages 6 and 10 years for boys and girls. Among boys, adequate screen time at age 2 years was associated with a 10% higher likelihood (*p* ≤ 0.05) of belonging to a persistent sport participation trajectory, in comparison to boys who had more than one hour of screen time per day. This association with sport participation trajectory was not significant among girls. Sleep and active leisure at 2 years did not significantly influence later sport participation for either boys or girls.

Among the confounding variables, for boys, lower maternal education reduced the likelihood of consistent sport participation by 33% (*p* ≤ 0.001). Additionally, maternal depressive symptoms forecasted an 11% increased likelihood of lower and inconsistent sport participation (*p* ≤ 0.01) years later, and insufficient family income predicted increases of 24% (*p* ≤ 0.001). For girls, lower family income, lower maternal education, more maternal depressive symptoms as well as BMI above the median reduced the likelihood of consistent sport participation by 20% (*p* ≤ 0.01), 19% (*p* ≤ 0.001), 11% (*p* ≤ 0.05), and 9% (*p* ≤ 0.01), respectively.

## 4. Discussion

Family environments play a central role in shaping long-term lifestyle patterns [10,34]. We examined naturally occurring parental choices regarding childhood lifestyle within a population-based framework. Our observations suggest that parental practices in toddlerhood can have lasting effects on child participation in organized sport.

Fewer than one in three boys spent less than an hour per day on screens as toddlers. That one-third was more likely to consistently participate in organized sport throughout middle childhood. This points to the importance of child-rearing practices that aim to regulate sedentary behavior early in life, as it may help lay the foundation for long-term physical activity. Such patterns in boys may be attributed to a combination of biological, psychological, and social factors. Biologically, boys tend to exhibit higher levels of motor activity and are consequently more sensitive to early constraints on movement, such as excessive screen use [35]. Psychologically and socially, boys are often encouraged, implicitly or explicitly, toward more active and competitive play than girls [23]. Parents may be more likely to limit screen time in favor of physical activity, aligning with broader societal norms that value athleticism in boys [36]. Notably, no consistent pattern in sport participation emerged when examining sleep and active leisure as features of early childhood lifestyle for boys.

Another plausible mechanism underlying the effects of screen time on child development may be differences in parenting styles and their influence on child activity levels [23]. Some parents actively regulate screen time and sedentary behaviors by encouraging alternative activities such as creative play, outdoor recreation, or organized sport. Parental regulation of screen time and encouragement of alternate activities emphasizes the importance of observational learning and role modelling.

In contrast, parents adopting more permissive or uninvolved approaches may impose fewer restrictions on screen time, potentially leading to prolonged sedentary behaviors. Excessive screen time exposure during early childhood may replace opportunities for motor skill development, which represents a critical predictor of future physical activity engagement [36]. This displacement effect might contribute to lower levels of physical literacy and negatively affect mental well-being by reducing time spent participating in more enriching and health-promoting activities [37].

Interestingly, screen time did not influence sport participation for girls, pointing to possible differences in how screen exposure affects development across sexes or how parental expectations around sport may vary for boys and girls [21,23]. According to ecological systems theory, these differences could reflect broader societal norms and gendered expectations around activity and sport that influence both parental decision-making and child opportunity [12]. For instance, societal norms might subtly encourage boys more towards activity and competitive sport while potentially placing less emphasis or even different expectations of female engagement in the same types of activities [36]. Such layered influences may help explain the distinct disruption of early screen time on sport participation trajectories in boys, but not in girls, underscoring the importance of considering sex-stratified developmental pathways in early childhood.

Given the strong influence of early rearing practices on later lifestyle, one would expect that engagement in active leisure during childhood would predict a consistent trajectory in organized sport. Surprisingly, contrary to expectation, our findings suggest that active leisure with parents at age 2 does not forecast sport participation in middle childhood. Active leisure could have been interpreted as reflecting parental values favoring physical activity, which might logically extend to organized sport. One possible explanation for this unexpected observation might be that some parents prioritize only active leisure to meet physical activity needs of their child, thus reducing the perceived necessity of organized sport participation. In this case, these parents could believe that unstructured physical activities, such as hiking, cycling or free play, offer comparable benefits to those provided by organized sport, thereby influencing their decision not to enroll them in later formal athletic programs [38]. These findings underscore the complexity of parental decision-making and highlight the importance of distinguishing between different types of physical activity when examining developmental trajectories of childhood movement.

Furthermore, the relationship between sleep duration and sport trajectory remains unclear. Logically, we might expect that children who sleep more have more energy and are therefore more likely to engage in physical activities, including organized sport. Adequate sleep is crucial for cognitive and physical functioning, which could facilitate greater engagement in sport [39]; however, this study did not find any association between meeting sleep recommendations in toddlerhood and consistent participation in middle childhood in sport. Further studies are needed to explore whether sleep directly influences long-term sport participation. Perhaps, sleep duration itself does not directly influence sport participation. However, there may be a threshold, below the recommended sleep duration, at which the effects of insufficient sleep might become more pronounced. When sleep falls beneath this threshold, it could lead to increased fatigue, diminished motivation, and impaired cognitive functioning, all of which could act as barriers to sport participation [40].

Although our findings did not reveal significant predictive effects of sleep duration or active leisure time on sport participation in middle childhood, this outcome underscores the multifactorial nature of parental decisions of child engagement in organized sport. Prior research highlights that beyond individual behaviors, broader contextual factors, particularly parental attitudes and values toward sport do play a crucial role in shaping participation patterns [11]. For instance, parents who prioritize physical activity and perceive sport as beneficial for health, discipline, or social development are more likely to enroll their children in organized sport programs, regardless of the child activity level or sleep routine [4,5,6,7,8]. Moreover, the informal and spontaneous nature of play-based activity may not always translate into structured sport participation, highlighting a distinction between intrinsic physical activity and externally organized forms.

Additionally, children’s involvement in organized sport tends to decline with age, suggesting that middle childhood may be a sensitive period where motivational, social, and environmental factors begin to exert a stronger influence [19]. These considerations point to the need for a more nuanced understanding of the ecological factors that support sport engagement.

Furthermore, our study identified several individual and family-related predictors of sport participation trajectories for both boys and girls. Specifically, both sons and daughters whose mothers had not completed high school, exhibited more depressive symptoms, or lived in lower-income households were less likely to participate in middle childhood sport. For daughters, mothers with a BMI above the median also emerged as a significant predictor of lower sport participation. As a result, children from such households have fewer opportunities to observe and internalize active lifestyle behaviors, such as monitoring screen time, establishment of regular sleep routines, and engagement in active leisure activities. These results align with previous research identifying these risk factors for increased sedentary behaviors and reduced engagement in sporting activities [4]. These same family and environmental factors are also associated with lower participation in organized sport among adults [41].

The present findings indicate that early restrictions on sedentary screen time for sons, as a proxy for parental structure and behavioral modeling, are prospectively associated with sport participation in middle childhood. This suggests that parenting behaviors during sensitive developmental windows may have enduring implications for the adoption of healthy parental choices and lifestyle habits by children [42]. Promoting screen time awareness among parents and caregivers could therefore serve as a key intervention point for supporting children long-term physical and psychosocial development.

Children sleep patterns, screen time, and engagement in active leisure appear to be shaped by a complex interplay of individual and family-level factors. Characteristics such as temperament, body weight, and parental mental health seem to influence how children develop routines, particularly around sleep and physical activity. For instance, higher BMI or difficulties in emotional regulation may contribute to irregular sleep durations and reduced physical engagement, possibly due to increased fatigue or lower motivation [43,44].

Family dynamics also play a crucial role in healthy habits. A less structured or more chaotic home environment, whether influenced by parental education, mental health, or socioeconomic status can limit opportunities for active play [45,46]. Also, it reinforces sedentary habits like screen use and disrupts consistent routines around sleep and movement behavior [47]. These patterns suggest that beyond individual traits, the broader caregiving context significantly affect the development of healthy behaviors.

Beyond the immediate family unit, access to affordable local programs, safe parks, and community centers significantly shapes child sport participation. For instance, in “sport deserts”, areas where such resources are lacking, even motivated parents may struggle to support involvement. Cost related to fees, equipment, and transport can further limit access, making sport participation not just on parenting, but on broader structural factors [46]. Early education settings like daycare and preschool can also play a key role in shaping sport involvement. Programs with active play, physical education, and outdoor spaces support motor skill development and physical literacy. Educators can model active behaviors, reinforcing, or compensating for varying parental emphasis on sport [48].

This study is not without limitations. This study uses data from a millennial birth cohort, capturing experiences that may differ from children today. However, considering the portability, plurality of screens, and concurrent use of multiple devices today, the measure used in this study captures total child screen time more accurately. Another limitation is the lack of detailed variables capturing parental involvement in sport, such as attitudes toward sport participation. This could provide valuable insights into the influence of family dynamics on sport participation.

Future research should delve deeper into parental motivations behind selecting child sporting activities and how this translates into intrinsic motivation for such venues in children. Understanding what genuinely drives desire to participate in children, whether it is innate enjoyment, a pursuit of mastery, or joy of social connection, is vital for identifying factors that sustain engagement and prevent attrition. Examining parental motivations and attitudes could similarly reveal how these values influence long-term participation trajectories.

One key strength of this study is its longitudinal design, which allows for the examination of developmental changes over time and provides a more naturalistic view of sport participation in childhood. Controlling pre-existing and confounding factors allowed for a more accurate and representative understanding of the relationship between early lifestyle habits and long-term outcomes. Additionally, stratifying analyses by sex reflects unique experiences and risk factors that boys and girls may encounter.

## 5. Conclusions

The disconcerting prevalence of sedentary behavior among youth underscores the critical importance of early childhood as a pivotal window for shaping lifelong healthy habits [49]. Beyond its physical health benefits, sport supports child cognitive development, emotional regulation, and social competence, with positive effects that often extend into adulthood [50]. This study reinforces the understanding that parental behaviors during these formative years significantly influence long-term behavior, particularly given the potential lasting impact of early screen time monitoring on child engagement with sport and physical activity. Ultimately, empowering families through targeted and accessible strategies, including education, community-based programming, and cultivating healthy daily habits from the earliest years of life will help mitigate the risks associated with long-term sedentary behavior.

## Figures and Tables

**Table 1:** Descriptive statistics for the variables in this study.

	Boys (%)	Girls (%)
***Predictors (age 2 years)***		
Screen time		
1 = below one hour a day	26.7	25.7
Sleep		
1 = between 11 to 14 h a day	78.1	78.1
Active leisure		
1 = at least once a day	33.3	35.0
Combined daily movement behaviors		
0 = don’t follow recommendation	11.9	11.1
1 = follow 1 recommendation	45.5	47.1
2 = follow 2 recommendations	35.2	33.7
3 = follow 3 recommendations	7.4	8.1
***Outcome (ages 6 to 10 years)***		
Extracurricular sport participation		
0 = low-inconsistent	37.6	34.2
1 = consistent	62.4	65.8
***Control variables***		
Maternal education (5 months)		
1 = did not finish high school	15.1	14.9
Maternal depression (5 months)		
1 = above the median	47.1	42.9
Child BMI (17 months)		
1 = above the median	50.6	45.7
Family dysfunction (17 months)		
1 = above the median	56.2	54.4
Maternal BMI (17 months)		
1 = above the median	50.9	51.2
Temperament problems (17 months)		
1 = above the median	50.7	51.0
Family configuration (2 years)		
1 = non-intact	14.1	12.0
Family income (2 years)		
1 = insufficient	17.8	17.8
Perceived physical activity (2 years)		
1 = less active than the others	2.3	3.1

Notes. BMI = Body Mass Index. Analyses corrected for attrition bias. Data compiled from the final master file of the Québec Longitudinal Study of Child Development (1998–2010), ©Government of Québec, *Institut de la statistique du Québec*.

**Table 2:** Adjusted unstandardized regression coefficients (with standard errors) reflecting the associations between pre-existing and concurrent individual and family characteristics from ages 5 months to 2 years, and movement behaviors at age 2 years, for boys and girls.

		Age 2 Years			
		Screen Time	Sleep	Active Leisure	Combined Daily MovementBehaviors
**Boys**	Maternal education (5 months)	−0.07 (0.06)	−0.08 (0.05)	0.01 (0.06)	−0.15 (0.10)
	Maternal depression (5 months)	−0.04 (0.04)	−0.04 (0.03)	−0.08 (0.04) *	−0.15 (0.06) *
	Child BMI (17 months)	0.00 (0.04)	−0.09 (0.03) **	0.03 (0.04)	−0.06 (0.06)
	Family dysfunction (17 months)	0.04 (0.04)	0.06 (0.03)	−0.02 (0.04)	0.08 (0.06)
	Maternal BMI (17 months)	−0.05 (0.04)	−0.02 (0.03)	0.02 (0.04)	−0.06 (0.06)
	Temperament problems (17 months)	−0.01 (0.04)	−0.07 (0.03) *	−0.00 (0.04)	−0.08 (0.06)
	Family configuration (2 years)	0.07 (0.08)	−0.02 (0.07)	−0.09 (0.08)	−0.03 (0.14)
	Family income (2 years)	−0.04 (0.06)	−0.06 (0.05)	−0.05 (0.06)	−0.14 (0.10)
	Perceived physical activity (2 years)	−0.17 (0.14)	0.04 (0.12)	0.13 (0.15)	0.00 (0.25)
	Adjusted R^2^	0.00	0.02 *	0.00	0.01 *
**Girls**	Maternal education (5 months)	−0.04 (0.05)	−0.15 (0.05) **	−0.01 (0.06)	−0.20 (0.10) *
	Maternal depression (5 months)	−0.09 (0.04) **	−0.03 (0.03)	−0.06 (0.04)	−0.19 (0.06)
	Child BMI (17 months)	−0.12 (0.03) ***	−0.01 (0.03)	−0.06 (0.04)	−0.18 (0.06) **
	Family dysfunction (17 months)	0.10 (0.04) **	0.02 (0.03)	−0.11 (0.04) **	0.01 (0.06)
	Maternal BMI (17 months)	−0.05 (0.03)	−0.01 (0.03)	0.00 (0.04)	−0.06 (0.06)
	Temperament problems (17 months)	0.00 (0.03)	0.01 (0.03)	−0.01 (0.04)	0.00 (0.06)
	Family configuration (2 years)	−0.01 (0.09)	−0.04 (0.08)	−0.17 (0.10)	−0.21 (0.17)
	Family income (2 years)	−0.13 (0.06) *	−0.11 (0.05) *	0.05 (0.06)	−0.19 (0.10) *
	Perceived physical activity (2 years)	−0.10 (0.10)	0.09 (0.09)	−0.11 (0.11)	−0.11 (0.18)
	Adjusted R^2^	0.04 **	0.02 *	0.02 *	0.04 ***

Notes. * *p* < 0.05, ** *p* < 0.01, *** *p* < 0.001. BMI = Body Mass Index. Analyses corrected for attrition bias. Data compiled from the final master file of the Québec Longitudinal Study of Child Development (1998-2010), ©Government of Québec, *Institut de la statistique du Québec*.

**Table 3:** Adjusted unstandardized regression coefficients (with standard errors) reflecting prospective associations between screen time, sleep, and active leisure at age 2 years and sport participation trajectories between ages 6 and 10 years, for boys and girls.

	Sport Participation Trajectory (Ages 6 to 10 Years)	
	Boys	Girls
*Predictors (age 2 years)*		
**Screen time**	−0.10 (0.05) *	**−0.00 (0.04)**
**Sleep**	**0.08 (0.05)**	**0.09 (0.06)**
**Active leisure**	**0.00 (0.05)**	**0.01 (0.05)**
*Control variables*		
Maternal education (5 months)	−0.32 (0.08) ***	−0.25 (0.06) ***
Maternal depression (5 months)	−0.11 (0.04) **	−0.11 (0.05) *
Child BMI (17 months)	0.01 (0.04)	−0.01 (0.04)
Family dysfunction (17 months)	−0.03 (0.04)	−0.03 (0.04)
Maternal BMI (17 months)	−0.01 (0.04)	−0.09 (0.04) **
Temperament problems (17 months)	−0.03 (0.04)	0.01 (0.04)
Family configuration (2 years)	−0.05 (0.16)	0.02 (0.11)
Family income (2 years)	−0.30 (0.06) ***	−0.25 (0.08) **
Perceived physical activity (2 years)	−0.15 (0.16)	0.04 (0.17)
Adjusted R^2^	0.13 ***	0.10 ***

Notes. * *p* < 0.05, ** *p* < 0.01, *** *p* < 0.001. BMI = Body Mass Index. Analyses corrected for attrition bias. Data compiled from the final master file of the Québec Longitudinal Study of Child Development (1998-2010), ©Government of Québec, *Institut de la statistique du Québec*.

## Data Availability

The data presented in this study are available on request from the *Institut de la Statistique du Québec* (ISQ). The data are not publicly available due to permission of the ISQ.
